# Enhanced Enzymatic Performance of β-Mannanase Immobilized on Calcium Alginate Beads for the Generation of Mannan Oligosaccharides

**DOI:** 10.3390/foods12163089

**Published:** 2023-08-17

**Authors:** Xinggang Chen, Zhuang Tian, Hongbo Zhou, Guoying Zhou, Haina Cheng

**Affiliations:** 1 Key Laboratory of National Forestry and Grassland Administration on Control of Artiffcial Forest Diseases and Pests in South China, Hunan Provincial Key Laboratory for Control of Forest Diseases and Pests, Key Laboratory of Cultivation and Protection for Non-Wood Forest Trees, Central South University of Forestry and Technology, Changsha 410004, China; xgchencsuft@163.com; 2 Key Laboratory of Biometallurgy, Ministry of Education, School of Minerals Processing and Bioengineering, Central South University, Changsha 410083, China

**Keywords:** alginate, mannanase, immobilization, kinetics, stability, mannan oligosaccharides

## Abstract

Mannan oligosaccharides (MOSs) are excellent prebiotics that are usually obtained via the enzymatic hydrolysis of mannan. In order to reduce the cost of preparing MOSs, immobilized enzymes that demonstrate good performance, require simple preparation, and are safe, inexpensive, and reusable must be developed urgently. In this study, β-mannanase was immobilized on calcium alginate (CaAlg). Under the optimal conditions of 320 U enzyme addition, 1.6% sodium alginate, 2% CaCl_2_, and 1 h of immobilization time, the immobilization yield reached 68.3%. The optimum temperature and pH for the immobilized β-mannanase (Man-CaAlg) were 75 °C and 6.0, respectively. The Man-CaAlg exhibited better thermal stability, a high degree of pH stability, and less substrate affinity than free β-mannanase. The Man-CaAlg could be reused eight times and retained 70.34% of its activity; additionally, the Man-CaAlg showed 58.17% activity after 30 days of storage. A total of 7.94 mg/mL of MOSs, with 4.94 mg/mL of mannobiose and 3.00 mg/mL of mannotriose, were generated in the oligosaccharide production assay. It is believed that this convenient and safe strategy has great potential in the important field of the use of immobilized β-mannanase for the production of mannan oligosaccharides.

## 1. Introduction

Mannan oligosaccharides (MOSs) are prebiotics mainly composed of 2–10 units of D-glucose and D-mannose connected by glycosidic bonds in which the main chain is connected by β-1,4 glycoside bonds and the branch chain is connected by a β-1,6 or β-1,3 glycosidic bond [1]. Studies have shown that they are widely used in medical applications, food and feedstuffs due to their anti-glycation function and their ability to improve intestinal microbial composition, lower blood sugar, lower blood lipids, reduce weight, improve immunity, and lower blood pressure [2,3,4]. According to statistics, the global prebiotics market size exceeded USD 2.90 billion in 2015 and will reach nearly USD 10.55 billion by 2025 [5]. Therefore, the generation of MOSs must meet the increasing demands of consumers.

Mannan, the raw material for the preparation of MOSs, comes from a variety of sources. Mannan is considered a kind of hemicellulose, which is widely found in konjac, coconut, palm, ivory nuts, coffee beans, locust beans, and other plants, as well as in the cell walls of fungi, bacteria, yeast, and other microorganisms [6]. However, the current production of oligosaccharides still cannot fully meet the needs of consumers, mainly due to limitations in preparation methods [7].

This prompted researchers to develop new oligosaccharide preparation methods. Some researchers use a combination of physical and biological enzymes to produce oligosaccharides [8,9,10]. Some researchers have developed more efficient enzymes [11,12]. Our research group has also developed an efficient β-mannanase that demonstrates activity up to 50,000 U/mL and strong thermal and acid stability [13,14]. However, the conventional enzymatic hydrolysis method also has some disadvantages: the enzyme cannot be reused, and it is not easy to separate the enzyme from the product, causing an increase in costs [15,16].

Immobilization is a prominent technology that enables enzymes to be reused, making the catalytic process more economically feasible and cost-effective [16]. It has been previously reported that β-mannanase was immobilized in sodium alginate or chitosan to improve its enzymatic properties [17,18]. In addition, Rahul et al. (2019) [19] immobilized a multi-enzymatic system (β-mannanase, β-glucosidase, endo-xylanase, β-xylosidase, and α-galactosidase) for the production of MOSs and juice processing [19]. However, except for the above, information on the use of immobilized β-mannanase to produce MOSs is scarce. Therefore, a simpler, safer, and more inexpensive immobilization method must be developed for the generation of MOSs.

**Figure 1:**
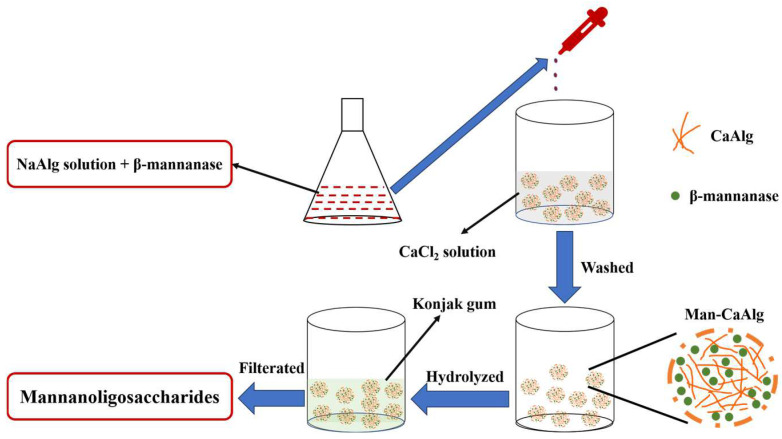
Systematic process for the preparation of Man-CaAlg and the generation of MOSs.

Various factors should be considered when using immobilized enzymes in industry, such as the selection of carrier materials that affect the efficiency and stability of the immobilized enzyme, which depends on the method of immobilization and the properties of the enzyme [20]. In addition, an immobilized enzyme and its carrier must be approved by the local Food and Drug Administration (FDA) before they can be used in the local food industry. Therefore, sodium alginate (NaAlg), a kind of natural polysaccharide, is an excellent carrier that meets the above conditions. It is widely used by immobilizers due to its easy availability, low cost, good biocompatibility, and ease of preparation [21]. In this study, biodegradable, inexpensive, and safe alginate and CaCl_2_ were used to immobilize β-mannanase, and the optimal immobilization conditions on the immobilized enzyme were determined. The properties of the immobilized enzyme and free enzyme were characterized. The enzyme demonstrated improved stability and activity after immobilization. MOSs were prepared from konjac gum by Man-CaAlg, and the yield of the MOSs was investigated (Figure 1). The ultimate aim of this research is to provide a simple, safe, and effective preparation method to reduce costs and increase yield.

## 2. Materials and Methods

### 2.1. Raw Materials and Chemicals

Konjac glucomannan (KGM) was purchased from Hefei BoMei Biotechnology Co., Ltd. (Hefei, China). The purity of the KGM was over 95%. Chemicals such as NaAlg, CaCl_2_, Na_2_HPO_4_, citric acid, 3,5-dinitrosalicylic acid (DNS), and similar were analytical grade and obtained from Sinopharm Chemical Reagent Co., Ltd. (Shanghai, China). NaAlg, Na_2_HPO_4_, citric acid, and CaCl_2_ were diluted with deionized water to obtain different concentrations. Different volumes of 0.2 mol/L Na_2_HPO_4_ and 0.1 mol/L citric acid were dissolved to obtain different pH Na_2_HPO_4_-citric acid buffers. The β-mannanase developed by our research group was purchased from Hunan Lerkam Biology Co., Ltd. (Changsha, China) [13,14].

### 2.2. Enzyme Assay

Protein concentration was determined by the Bradford method [22]. The activity of β-mannanase was estimated using 0.5% (*w*/*v*) locust bean gum (LBG) as the substrate by the DNS method [23]. The procedure for measuring enzyme activity was as follows: 0.9 mL of LBG was incubated with 0.1 g of Man-CaAlg or 0.1 mL of diluted β-mannanase for 15 min. Subsequently, 1.5 mL of DNS was immediately added to the reaction and boiled for 5 min to stop the reaction. Reducing sugar was detected at 540 nm, and mannose was used as standard [24]. One unit of β-mannanase activity was defined as the amount of β-mannanase that produced 1 μmol of reducing sugars per min.

(1) $$\mathrm{Immobilization}\;\mathrm{yield}\;(\%)=\frac{\mathrm{Specific}\mathrm{activity}\mathrm{of}\mathrm{immobilized}\beta \text{-}mannanase}{Specific\;activity\;of\;free\;\beta \text{-}mannanase}\times 100$$

(2) $$\mathrm{Specific}\;\mathrm{activity}\;\mathrm{of}\;\mathrm{free}\;\beta \text{-}mannanase\;(U/\mathrm{mg})=\frac{A}{B}$$

(3) $$\mathrm{Specific}\;\mathrm{activity}\;\mathrm{of}\;\mathrm{immobilized}\;\beta \text{-}mannanase\;(U/\mathrm{mg})=\frac{A-C}{B-D}$$

where A is the total activity for immobilization, B is the total protein for immobilization, C is the total activity remaining in the supernatant after immobilization, and D is the total protein remaining in the supernatant after immobilization.

### 2.3. Effect of Immobilization Conditions on β-Mannanase

The procedure for immobilizing β-mannanase was as follows: an appropriate amount of NaAlg and β-mannanase were dissolved in deionized water together. The mixture was dropped into an appropriate concentration of CaCl_2_ solution dissolved in deionized water. The formed calcium alginate (CaAlg) gel beads were washed with deionized water. The washed gel bead was the Man-CaAlg.

Effect of enzyme addition on β-mannanase: 40, 80, 160, 240, 320, 480, and 640 U of β-mannanase solution (1 mL) was added into 100 mL 2% (*w*/*v*) NaAlg solution and then the mixture was dropped into 2% (*w*/*v*) CaCl_2_ solution for 1 h to obtain the immobilized enzyme gel beads.

Effect of concentration of NaAlg on β-mannanase: 240 U of β-mannanase solution (1 mL) was added into 100 mL of different concentrations (1.2, 1.6, 2, 2.4, and 2.8%) of NaAlg solution and then the mixture was dropped into 2% CaCl_2_ solution for 1 h to obtain the immobilized enzyme gel beads.

Effect of concentration of CaCl_2_ on β-mannanase: 240 U of β-mannanase solution (1 mL) was added into 100 mL 2% NaAlg solution and then the mixture was dropped into 1, 2, 4, 6, and 8% CaCl_2_ solution for 1 h to obtain the immobilized enzyme gel beads.

Effect of immobilization time on β-mannanase: 240 U of β-mannanase solution (1 mL) was added into 100 mL 2% NaAlg solution and then the mixture was dropped into 2% CaCl_2_ solution for 0.25, 0.5, 0.75, 1, 2, and 4 h to obtain the immobilized enzyme gel beads.

### 2.4. Effect of Temperature and pH of Man-CaAlg

The activities of free β-mannanase and Man-CaAlg were measured at a range of pH 4.5 to 8.0 to find the optimal pH using the Na_2_HPO_4_-citric acid buffer. The activities of Man-CaAlg and free β-mannanase were tested at different temperatures (40 to 90 °C) to determine the optimal temperature. Residual β-mannanase and Man-CaAlg activities were measured after pre-treatment at different temperatures (60–90 °C) or pH (4.0–8.0) for 30 min to determine the temperature or pH stability of free β-mannanase and Man-CaAlg. The highest activity of enzymes in each set was designated as 100% activity. The activity of the enzymes was obtained by the percentage of enzyme activity to the highest enzyme activity.

### 2.5. Determination of Kinetic Parameters

The enzyme reaction rate is influenced by the substrate concentration of that particular enzyme. This relationship follows Michaelis–Menten kinetics listed in the following equation:

(4) $$\frac{1}{V}=\frac{K_{m}}{V_{m}\left[S\right]}+\frac{1}{V_{m}}$$

where V_m_ is the maximum reaction rate, V is the reaction rate of the enzyme, [S] is the substrate concentration, and K_m_ is the Michaelis–Menten constant. The V_m_ and K_m_ were measured through a Lineweaver–Burk plot [25] of 1/V versus 1/S, at different substrate (LBG) concentration of 1–5 mg/mL prepared in Na_2_HPO_4_–citric acid buffer (pH 5.5), and enzyme assay was performed.

### 2.6. Reusability and Storage Stability of Man-CaAlg

The attenuation of Man-CaAlg activity was measured as described in Section 2.2. After each cycle, the Man-CaAlg was washed with deionized water to remove residual substrates (LBG). Further, the washed Man-CaAlg reacted with the fresh substrate (LBG) under the same conditions to determine the enzyme activity. These steps were cycled 8 times.

Both free β-mannanase and Man-CaAlg were placed in deionized water and stored at 4 °C. The relative activity of Man-CaAlg and free β-mannanase was measured every 5 days. The fresh Man-CaAlg and free β-mannanase were considered to be 100% activity.

### 2.7. Enzymatic Hydrolysis of KGM

The hydrolysis of KGM to produce MOSs was carried out by Man CaAlg. Approximately 1 L KGM gum (2% *w*/*v* in Na_2_HPO_4_–citric acid buffer, pH 6.0) was incubated with Man-CaAlg (50 U/mL) in a column for 8 h at 75 °C and 150 rpm. The hydrolysate was placed in boiling water for 15 min to inactivate the free β-mannanase leaked from Man CaAlg and then centrifuged at 1000× *g* for 15 min to precipitate the insoluble substance. Thereafter, the hydrolysate was stored at −20 °C for subsequent analysis.

The hydrolysate was analyzed by high-performance liquid chromatography (HPLC) LC-20A purchased from Shimadzu (Shanghai, China) Global Laboratory Consumables Co., Ltd. (Shanghai, China) with an evaporative light scattering detector ELSD-2000ES purchased from Alltech Technology Co., Ltd. (Chicago, IL, USA). The analysis was completed on an Athena NH_2_ column (250 × 4.6 mm internal diameter, 5 µm particle size, 120 Å) purchased from ANPEL Laboratory Technologies Co., Ltd. (Shanghai, China). with 100 mmol/L NaOH as the mobile phase at a flow rate of 1.0 mL/min. The temperature of the column was maintained at 25 °C.

(5) $$Lgc_{1}=\frac{Lgc_{2}\times LgS_{1}}{LgS_{2}}$$

where c_1_ is the sugar concentration of a peak in the hydrolysate, c_2_ is the concentration of mannobiose standard, S_1_ is the peak area corresponding to a peak in the hydrolysate, and S_2_ is the peak area of mannobiose standard.

### 2.8. Statistical Analysis

All of the experiments were performed in triplicate and the results were described as average ± standard deviation (SD). The bars followed by different lowercase letters were significantly different at *p* < 0.05 using ANOVA. All statistical analyses of the data were carried out by Excel 2019 purchased from Microsoft (China) Co., Ltd. (Beijing, China), and the figures were exported by Origin2021 purchased from OriginLab Corporation (Northampton, UK).

## 3. Results and Discussion

### 3.1. Effect of Immobilization Conditions on Man-CaAlg

The effect of different immobilization conditions on the immobilization yield is very significant. Therefore, we selected the four most important factors, the enzyme addition (U), the concentration of NaAlg (%), the concentration of CaCl_2_ (%), and the immobilization time (h) to explore their effect on the immobilization yield.

**Figure 2:**
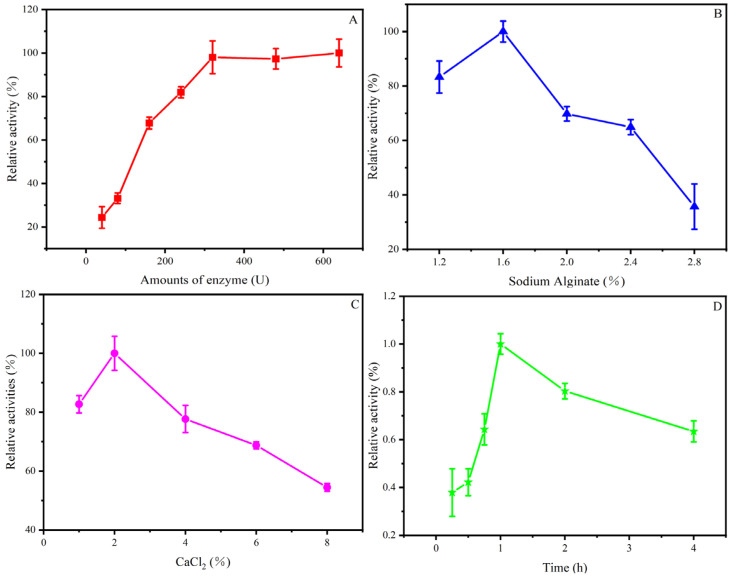
The immobilization of β-mannanase on calcium alginate beads as affected by the amounts of enzyme (**A**), NaAlg concentration (**B**), CaCl_2_ concentration (**C**), and immobilization time (**D**).

As shown in Figure 2A, with the increase in enzyme addition, the relative activity of Man-CaAlg continued to increase until the enzyme addition exceeded 320 U, and the enzyme activity did not increase significantly. When the amount of added enzyme was less than 320 U, since the NaAlg microspheres had sufficient capacity to intercalate β-mannanase, the activity of Man-CaAlg rapidly increased. When the amount of enzyme added was more than 320 U, the Man-CaAlg activity increased slowly, which may be caused by NaAlg hindering the binding of β-mannanase to the substrate (Figure 2A). Another reason probably was that the space in NaAlg that can encapsulate β-mannanase was limited. If the amount of β-mannanase is increased, it will only leak out and cannot be fixed in NaAlg microspheres [26]. Therefore, the enzyme addition of 320 U was selected for subsequent assay.

In order to improve the performance of Man-CaAlg, the optimal concentration of NaAlg needs to be determined so that the product and substrate can pass through the calcium alginate (CaAlg) gel beads easily without β-mannanase leakage from the gel beads. Preventing β-mannanase leakage from CaAlg gel beads is quite difficult. However, its leakage can be prevented by increasing the concentration of NaAlg. Therefore, selecting the optimal concentration of NaAlg is essential for the diffusion of the substrate to CaAlg gel beads and the immobilization of β-mannanase as the porosity of support gel beads is basically determined by the concentration of NaAlg. In addition, the concentration of NaAlg also plays a significant role in immobilization by affecting various properties such as bead strength, swelling, stability, and porosity [27,28]. Too high a concentration of NaAlg will cause difficulty in combining the enzyme with the substrate, while too low a concentration of NaAlg will cause the leakage of the enzyme. As shown in Figure 2B, with the increasing concentration of NaAlg, the relative activity of Man-CaAlg showed a trend of increasing first and then decreasing. When the concentration of NaAlg was lower than 1.6%, the relative activity increased continuously. When the concentration of NaAlg was higher than 1.6%, the relative activity decreased gradually (Figure 2B). Therefore, it was found that a 1.6% NaAlg concentration was the most appropriate.

Since CaCl_2_ can lead to the deactivation of β-mannanase, it is very important to determine the optimal concentration of CaCl_2_ and the immobilization time. Too low a CaCl_2_ concentration or too short an immobilization time will lead to insufficient immobilization of β-mannanase, while too high a CaCl_2_ concentration or too long an immobilization time will lead to the deactivation of β-mannanase. As shown in Figure 2C, when the concentration of CaCl_2_ was lower than 2%, the pores of the NaAlg gel beads were too large, and it was easy for β-mannanase to pass through the pores and leak from the CaAlg gel beads. When the concentration of CaCl_2_ was higher than 2%, the pores of the CaAlg gel beads were too small, which was not conducive to the combination of substrate and enzyme, and the high concentration of CaCl_2_ led to the inactivation of β-mannanase, which ultimately led to a decrease in the relative activity of Man-CaAlg with the increase in CaCl_2_ concentration. Therefore, the concentration of 2% CaCl_2_ was selected for subsequent assay.

By the same token, when the immobilization time was less than 1 h, β-mannanase could not be fully encapsulated (Figure 2D). Therefore, with the increase in the immobilization time, the relative activity of Man-CaAlg increased gradually. When the immobilization time exceeded 1 h, the CaAlg gel beads formed too dense pores, which blocked the combination of β-mannanase and the substrate and led to the inactivation of β-mannanase. Therefore, the relative activity of Man-CaAlg decreased with the prolongation of the immobilization time. Finally, the immobilization time of 1 h was selected for subsequent assay. We chose the above optimal conditions to immobilize β-mannanase, and the results showed that the immobilization yield was 68.3% (Table 1). Dhiman et al. (2020) [29] successfully immobilized mannanase on sodium alginate-grafted-β-cyclodextrin and the immobilization yield reached 91.5%. However, its preparation steps were more complex, and more reagents such as cyclodextrin were used. Moreover, the use of hazardous chemicals such as NaOH posed a risk of danger during preparation. Therefore, a safer and simpler preparation step is the advantage of this study [29].

### 3.2. Effect of Temperature and pH on Man-CaAlg and Free β-Mannanase

**Figure 3:**
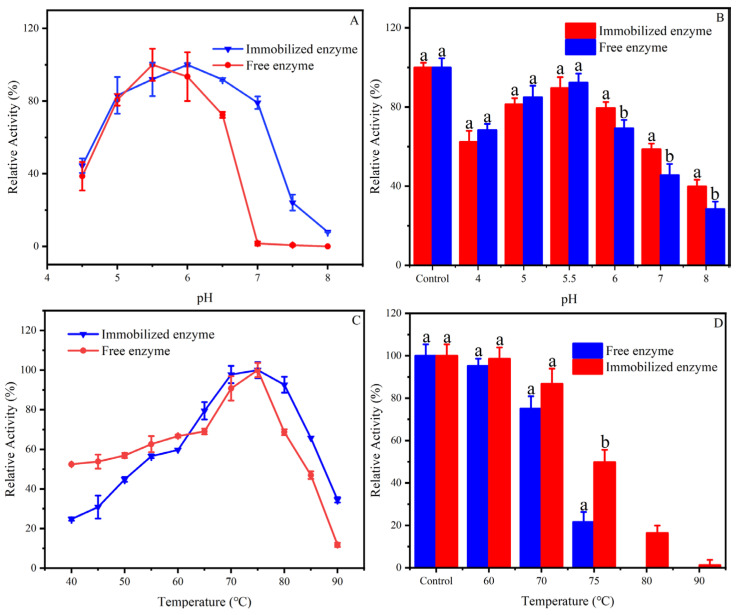
Optimal pH (**A**), pH stability (**B**), optimal temperature (**C**), and thermostability (**D**) of Man-CaAlg. The error bars are described the as average ± standard deviation (SD). The bars followed by different lowercase letters were significantly different at *p* < 0.05 using an ANOVA.

The activity and stability of enzymes at different temperatures and pHs are of great significance for industrial applications. In this study, the effect of different pH values (4–8) on the relative activity in free β-mannanase and Man-CaAlg was evaluated. As can be seen from Figure 3A, with the increase in the pH value, the relative activity of Man-CaAlg and free β-mannanase showed a trend of increasing first and then decreasing. When the pH value was lower than 5.5, the relative activity of free β-mannanase gradually increased, and when the pH value was higher than 5.5, the relative activity of free β-mannanase gradually decreased. Similarly, when the pH value was lower than 6.0, the relative activity of Man-CaAlg gradually increased, and when the pH value was higher than 6.0, the relative activity of Man-CaAlg gradually decreased (Figure 3A). Therefore, it can be seen from Figure 3A that the optimum pH of Man-CaAlg shifted from 5.5 which was the optimum for free β-mannanase to 6.0. Moreover, when the pH was more than 5.5, the Man-CaAlg showed better activity, which was similar to the previous report (Figure 3A) [18]. When the pH value was 7, free β-mannanase was inactivated, while Man-CaAlg still maintained 79.1% relative activity.

In order to explore the pH stability of Man-CaAlg and free β-mannanase, the Man-CaAlg and free β-mannanase were placed at different pHs for 30 min, and then the residual activity was measured at optimum pH. The results are shown in Figure 3B, the Man-CaAlg showed better stability at pH 6, 7, and 8, retaining 79.48, 58.63 and 39.82% residual activity, respectively, while free β-mannanase retained only 69.26, 45.55, and 28.46% residual activity, respectively. This slight change in pH could be attributed to the substantial difference in the ionic environment of the carrier around the enzyme active sites. In the preparation of MOSs by β-mannanase, ions must be added to adjust the pH, which will cause trouble in the purification of the MOSs. However, the improved high pH stability of Man-CaAlg makes it possible to omit the pH adjustment in production.

The effect of different temperatures (40–90 °C) on the relative activity in free β-mannanase and Man-CaAlg was evaluated. As can be seen from Figure 3C, when the temperature was between 40 and 90 °C, the relative activities of Man-CaAlg and free β-mannanase increased first and then decreased with the increase in temperature. When the temperature was lower than 75 °C, the relative activity of free β-mannanase and Man-CaAlg gradually increased, and when the temperature value was higher than 75 °C, the relative activity of free β-mannanase and Man-CaAlg gradually decreased (Figure 3C). Therefore, it can be seen from Figure 3C that after the immobilization of β-mannanase, the optimum temperature remained unchanged at 75 °C. However, Man-CaAlg showed better activity at 80, 85, and 90 °C, retaining 92.6, 65.7 and 34.5% relative activity, respectively, while free β-mannanase retained only 68.7, 47.0, and 11.8% relative activity, respectively (Figure 3C). Therefore, when the temperature was higher than 75 °C, the relative activity of Man-CaAlg was higher than that of β-mannanase. The thermostability of Man-CaAlg may be better than that of β-mannanase.

In order to verify that the thermostability of Man-CaAlg was better than that of free β-Mannanase, the Man-CaAlg and free β-Mannanase were placed at different temperatures for 30 min, and then the remaining activity was measured at optimum temperature. The results are shown in Figure 3D. At 60 °C, Man-CaAlg and free mannanase showed similar stability. However, Man-CaAlg showed better stability at 70–90 °C. After incubation at 70 and 75 °C, the Man-CaAlg retained 86.76% and 49.83% residual activity, respectively, while the free enzyme retained only 75.12% and 21.66% residual activity, respectively. Moreover, the Man-CaAlg retained 16.4% residual activity at 80 °C, while the free β-mannanase had been inactivated (Figure 3D). Therefore, the thermostability of Man-CaAlg was superior to that of free β-mannanase. Previous reports also show that immobilization increased the thermostability and half-life of the enzyme when compared to the free enzyme [30]. It was observed that the immobilization process on NaAlg protected the β-mannanase against heat inactivation. Such enhancements in the thermal resistance of Man-CaAlg over the free enzyme agreed with some of the previous reports [30,31]. The increase in thermostability might arise from the thermal barrier effect of NaAlg on β-mannanase, and the support and protection effect of NaAlg on the active center of β-mannanase. To sum up, its good thermal stability makes it possible for this immobilized enzyme to be used in industry.

### 3.3. Enzyme Kinetics of Man-CaAlg and Free β-Mannanase

**Figure 4:**
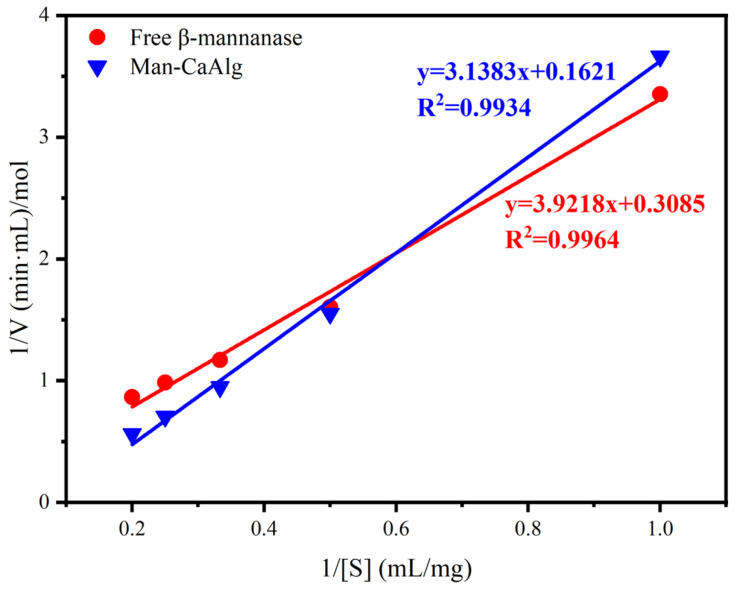
Kinetic parameters of free β-mannanase and Man-CaAlg determined by a Lineweaver–Burk plot.

The quality of immobilization was estimated by measuring changes in the kinetic parameters. The rate of reaction and affinity of Man-CaAlg or free β-mannanase towards the substrate (LBG) will be indicated by V_max_ and K_m_, respectively. The K_m_ and V_max_ of the free β-mannanase and Man-CaAlg were evaluated by using the substrate LBG at different concentrations. Figure 4 shows the Lineweaver–Burk plots of free β-mannanase and Man-CaAlg. The K_m_ value was found to be 10.17 and 19.36 mg/mL for the free β-mannanase and Man-CaAlg, respectively. The higher the K_m_ value of Man-CaAlg, the lower the affinity of Man-CaAlg to the substrate, which may be caused by the increase in the steric hindrance of the active site and the loss of flexibility of the enzyme necessary for substrate binding [32]. Previous studies have also shown that the affinity of most immobilized enzymes to substrates will be weakened [33]. Moreover, the V_max_ of Man-CaAlg increased from 3.24 to 6.17, which may be due to improper conformation of the enzyme in the CaAlg gel beads. Previous studies also report that the K_m_ and V_max_ of the free enzyme (LXy) were lower than the immobilized enzyme (LXy-NaAlg), suggesting that the free enzyme has a high affinity to the substrate [34]. In addition, the enhancement of the affinity of the immobilized enzyme and a reduction in the reaction rate were also commonly reported [35,36,37]. These results clearly indicate that the immobilization of β-mannanase on CaAlg gel beads reduces the affinity between the β-mannanase and the substrate, which is due to a reduction in the contact area between the substrate and the β-mannanase.

### 3.4. Reusability and Storage Stability of Man-CaAlg

**Figure 5:**
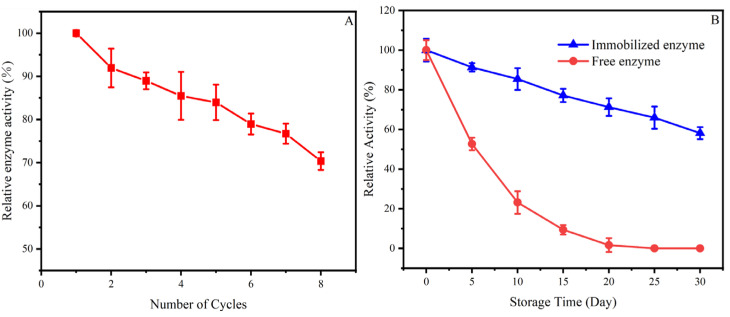
Reusability of Man-CaAlg (**A**) and storage stability of Man-CaAlg and free β-mannanase (**B**). The error bars are described as the average ± standard deviation (SD).

The reusability of immobilized enzymes is the top priority in industrial applications due to its influence on operational stability [38]. The results of Man-CaAlg activity repeatedly measured eight times under the optimal conditions are shown in Figure 5A. As the number of cycles increased, the activity of Man-CaAlg gradually decreased. However, Man CaAlg maintained activity of 70.34% and good reusability after 8 cycles. Similar results were obtained for other enzymes immobilized on sodium alginate [39,40]. This may be due to enzyme denaturation caused by repeated heating [30]. Moreover, according to Yang et al. (2014)’s report, the reusability of immobilized *Candida rugosa* Lipase (CRL) is better than that of immobilized *Brassica oleracea* Chlorophyllase 1 (BoCLH1), even though their carriers are the same [41]. Therefore, the reusability of the immobilized enzyme is also related to the property of the enzyme itself.

The storage stability of immobilized enzymes is crucial for industrial applications. Both Man-CaAlg and free β-mannanase were placed in deionized water and stored at 4 °C for 30 days, and the enzyme activity was measured every 5 days. Figure 5B shows that with the increase in storage time, the activity of both Man-CaAlg and free β-mannanase decreased gradually. However, at the same storage time, the activity loss of Man-CaAlg was much less than that of free β-mannanase. When the storage time was 5 days, the activity of free β-mannanase was only about half, while that of Man-CaAlg was 91.32%. When the storage time was 15 days, the relative activity of free mannanase was only 9.41%, almost inactivated, while the activity of Man-CaAlg remained at 77.15%. After 25 or 30 days of storage, Man-CaAlg retained 65.97 or 58.17% relative activity, while the free enzyme lost its activity. This clearly shows that Man-CaAlg is more stable and advantageous for industrial processes. This might be associated with the minimal distortion of the enzyme active sites caused by the immobilization, which prevented the inactivation of β-mannanase, resulting in higher storage stability [42].

### 3.5. MOSs Generation

**Figure 6:**
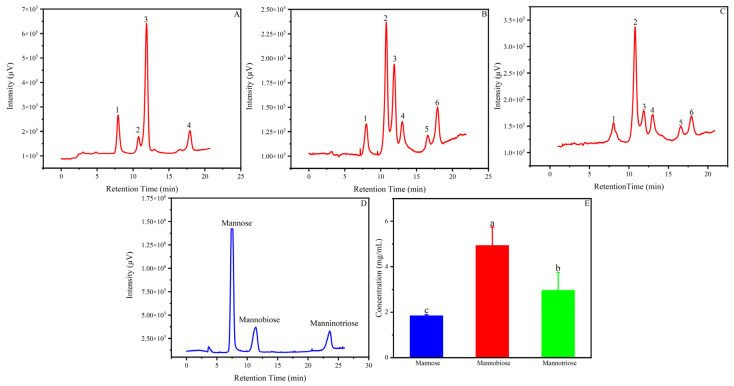
End product analysis of KGM hydrolysis by Man-CaAlg. HPLC of No. 1 KGM hydrolysate. Peaks 1, 2, 3, and 4 represent mannose, mannobiose, mannobiose and mannotriose, respectively (**A**). HPLC of No. 2 KGM hydrolysate. Peaks 1, 2, 3, 4, 5, and 6 represented mannose, mannobiose, mannobiose, mannobiose, mannotriose, and mannotriose (**B**). HPLC of No. 3 KGM hydrolysate. Peaks 1, 2, 3, 4, 5, and 6 represented mannose, mannobiose, mannobiose, mannobiose, mannotriose, and mannotriose (**C**). HPLC of standards (mannose, mannobiose, and mannotriose) (**D**). Yield of MOSs (**E**). The error bars are described as the average ± standard deviation (SD). The bars followed by different lowercase letters were significantly different at *p* < 0.05 using ANOVA.

To investigate the production potential of Man-CaAlg, mannooligosaccharide production assays were performed. Monia reported that the immobilized *Penicillium occitanis* mannanase did not hydrolyze after 8 h [18]. Therefore, we chose a hydrolysate with a hydrolysis time of 8 h for HPLC analysis to explore the yield of MOSs. As shown in Figure 6D, after 8 h, a total of 7.94 mg/mL MOSs with 4.94 mg/mL of mannobiose and 3.00 mg/mL of mannotriose, respectively, were generated. In addition, 1.84 mg/mL of mannose was generated. MOSs generation from KGM hydrolysis was 39.7% (*w*/*w*) which indicated that Man-CaAlg worked well.

## 4. Conclusions

In this study, β-mannanase was simply, efficiently, and safely immobilized on CaAlg gel beads to improve performance. The results showed that the immobilization yield of Man-CaAlg was 68.3% using optimized conditions (320 U enzyme addition, 1.6% NaAlg, 2% CaCl_2_, and 1 h immobilization time). Furthermore, Man-CaAlg exhibited better high pH and temperature stability and exhibited the highest activity at 75 °C and pH 6.0. However, the kinetic parameters showed that Man-CaAlg has slightly less affinity for the substrate than the free β-mannanase. Subsequently, Man-CaAlg exhibited excellent storage stability and acceptable reusability. A total of 7.94 mg/mL MOSs, with 4.94 mg/mL of mannobiose and 3.00 mg/mL of mannotriose, were generated in the MOS production assays, indicating the excellent production potential of Man-CaAlg. Herein, the results suggest that Man-CaAlg enhanced enzymatic performance and that it can be used as an effective, inexpensive, reusable, and safe catalyzer for MOSc generation.

## Figures and Tables

**Table 1:** Immobilization yield of Man-Alg using optimized conditions.

Enzyme Form	Specific Activity (U/mg)	Immobilization Yield (%)
Free β-mannanase	15.4 ± 2.2	
Man-CaAlg	22.5 ± 1.5	68.3%

## Data Availability

All data included in this study are available upon request by contacting the corresponding author.
